# Default network interactivity during mentalizing about known others is modulated by age and social closeness

**DOI:** 10.1093/scan/nsaa067

**Published:** 2020-05-13

**Authors:** Anne C Laurita, Elizabeth DuPre, Natalie C Ebner, Gary R Turner, R Nathan Spreng

**Affiliations:** 1 Health Promotion & Prevention Services, University Health Services, Princeton University, Princeton, NJ 08544, USA; 2 Department of Neurology and Neurosurgery, Montreal Neurological Institute, McGill University, Quebec H3A 2B4, Canada; 3 Department of Psychology, University of Florida, Gainesville, FL 32611, USA; 4 Department of Aging and Geriatric Research, Institute on Aging, University of Florida, Gainesville, FL 32611, USA; 5 Department of Clinical and Health Psychology, Center for Cognitive Aging and Memory, University of Florida, Gainesville, FL 32611, USA; 6 Department of Psychology, York University, Toronto, ON M3J 1P3, Canada; 7 Department of Psychiatry, McGill University, Quebec H3A 2B4, Canada; 8 Department of Psychology, McGill University, Quebec H3A 2B4, Canada; 9 McConnell Brain Imaging Centre, Montreal Neurological Institute, McGill University, Quebec H3A 2B4, Canada

**Keywords:** aging, social cognition, mental representations, default network, fMRI, functional connectivity, dedifferentiation, default-executive coupling, DECHA

## Abstract

In young adults, mentalizing about known others engages the default network, with differential brain response modulated by social closeness. While the functional integrity of the default network changes with age, few studies have investigated how these changes impact the representation of known others, across levels of closeness. Young (*N* = 29, 16 females) and older (*N* = 27, 12 females) adults underwent functional magnetic resonance imaging (fMRI) scanning while making trait judgments for social others varying in closeness. Multivariate analyses (partial least squares) identified default network activation for trait judgments across both age cohorts. For young adults, romantic partner and self-judgments differed from other levels of social closeness and were associated with activity in default and salience networks. In contrast, default network interactivity was not modulated by social closeness for older adults. In two functional connectivity analyses, both age groups demonstrated connectivity between dorsal and ventral medial prefrontal cortex and other default network regions during trait judgments. However older, but not young, adults also showed increased functional coupling between medial and lateral prefrontal brain regions that did not vary by category of known other. Mentalizing about others engages default and frontal brain regions in older adulthood, and this coupling is poorly modulated by social closeness.

## Introduction

Throughout the life course, we constantly form, update and use representations of social others. Human social environments comprise complex hierarchies and feature a wide spectrum of relationships, ranging in proximity from close others and attachment figures to more distant acquaintances. As close social relationships have been shown to confer various psychological and physiological benefits (Carstensen *et al.,* 1996; Hoppmann and Gerstorf, 2009), it is perhaps one of our most crucial human cognitive capacities to be able to differentiate representations of close from less close others in our social world, throughout the adult lifespan.

Social cognition, and specifically our capacity to form and access representations of close others, has been associated with multiple brain systems (for review, see Laurita *et al.,* 2019b). Social and evolutionary psychological theories have emphasized the highly rewarding and functionally adaptive nature of close social bonds, implicating the brain’s reward and distress alleviation systems (e.g. Bartels and Zeki, 2004; Coan *et al.,* 2006; Acevedo *et al*., 2012). In contrast, cognitive psychological theories have focused on the specific processes through which representations of social others are formed, stored and accessed, investigating how these processes and representations are implemented in the brain (e.g. Heatherton *et al.,* 2006; Krienen *et al.,* 2010; Beckes *et al.,* 2013). This latter line of research, emphasizing the processing of social representations, has implicated a number of brain areas in mentalizing or attending to the mental state or characteristics of known others. These regions of the ‘social brain’ (Mitchell, 2008) closely overlap with the default network, a functionally connected assembly of brain regions that has been associated with internal mentation and social cognitive processing (for review, see Spreng and Andrews-Hanna, 2015).

The default network is composed of areas along the cortical midline, including dorsal medial prefrontal cortex (dmPFC), ventral medial prefrontal cortex (vmPFC) and posterior cingulate cortex (PCC), as well as medial and lateral temporal cortices, lateral parietal lobes and caudal portions of the lateral prefrontal cortex (Andrews-Hanna *et al.,* 2007; Buckner *et al*., 2008). Together, these regions have been implicated in our ability to imagine the experiences of others (Spreng *et al.,* 2009; Hassabis *et al.,* 2014), attribute and judge emotional states (Haas *et al.,* 2015), reflect on beliefs (Young *et al.,* 2010) and form social impressions of known others (Cassidy and Gutchess, 2012).

Social representations are stratified along multiple dimensions, including personal similarity (Mitchell *et al.,* 2006), relational hierarchy (Tavares *et al.,* 2015) and social proximity or ‘closeness’ (Krienen *et al.,* 2010). These various dimensions of social relatedness differentially impact how representations of social others are implemented in the brain (Thornton and Mitchell, 2017). In this context, social proximity (or closeness) in particular has been strongly implicated in the differential recruitment of the default network (Gobbini *et al.,* 2004; Krienen *et al.,* 2010). Further, there is evidence that interactivity between default brain regions and other neural networks may be critical in the stratification of one’s social world. For example, interactions between the default network and the medial temporal lobe memory system have been shown to support navigation of, or tracking shifting dynamics in and responding to, interpersonal relationships based on social affiliation and power hierarchies (Tavares *et al.,* 2015). Similarly, we have shown interactions between the default network and brain regions implicated in detecting personally salient stimuli (i.e. salience network; Uddin, 2015) during trait judgments of self and romantic partners (Laurita *et al.,* 2017), but not for more socially distant others, as will be addressed in the present paper.

While much of the existing research investigating neural representations of social others has relied on young adult cohorts, few studies have examined this process in older adults (e.g. Ebner *et al.,* 2011, 2013). Various social cognitive abilities change across the adult lifespan, with evidence of both gains and losses (see Ebner *et al.,* 2016 for a review). Within the domain of mentalizing about social others, the evidence for age-related change is equivocal and may depend on the specific nature of the social judgment required (Reiter *et al.,* 2017). In one neuroimaging investigation, older adults performed more poorly than young adults on assessments of moral judgment and false beliefs (Moran *et al.,* 2012). In contrast, Castelli *et al*. (2010) reported comparable performance for older and young adults on a theory of mind task. As mentalizing about social others depends on fluid intellectual or executive control processes (German and Hehman, 2006; Bailey *et al.,* 2008; Charlton *et al.,* 2009), age-related declines in cognitive control may contribute to reduced social cognition in later life, although this idea remains controversial (Maylor *et al.,* 2002; Sullivan and Ruffman, 2004). A comprehensive meta-analytic review found that older adults have reliable difficulty in performing theory of mind tasks regardless of specific task parameters, with deficits across task type, domain and modality (Henry *et al.,* 2013).

At the level of the brain, regions implicated in social functioning closely overlap with the default network (Mitchell, 2008). The default network undergoes significant change in normal aging, with evidence of reduced within-network and increased between-network connectivity (see Damoiseaux, 2017 for a review). It seems plausible then that these age-related changes may be associated with differences in mentalizing abilities between young and older adults. Only a few studies, however, have investigated this possibility directly. Moran *et al*. (2012) reported that activity in a core node of the default network, dmPFC, was reduced in older *vs* young adults during a series of social cognitive tasks involving inferences about the intents, actions and mental states of others. In another investigation, activity in dmPFC as well as other core default network nodes, including vmPFC and PCC, was reduced during negative (and increased during positive) impression formation in older *vs* young adults (Cassidy *et al*., 2013). In more recent work, medial PFC activity was lower for older *vs* younger adults during a face perception mentalizing task (Cassidy *et al.,* 2020). Lower resting state functional connectivity within the default network has also been associated with poorer performance on a theory of mind task in older adults (Hughes *et al.,* 2019). In contrast, an earlier study reported no age differences in activation or functional connectivity of the default network during a mentalizing task (Castelli *et al.,* 2010). As noted above, older adult performance was comparable to young in this study, prompting the authors to speculate that greater recruitment of lateral PFC regions may have supported mentalizing ability in the older adults.

Taken together, research suggests that for young adults, the default network is implicated in social cognitive abilities, including mentalizing about social others. Further, the magnitude and topological pattern of default network engagement is modulated by social closeness. Despite well-documented changes in the default network with age, relatively little is currently known about how age-related differences in the default network relate to age-related differences in mentalizing, and no study to date has examined variations in these effects across levels of closeness among known social others. Here we use functional magnetic resonance imaging (fMRI) to examine the neural representations of mentalizing about known others in young and older adults across a continuum of social closeness. Extending our recent study of young adults (Laurita *et al.,* 2017), we used a trait judgment task to assess mentalizing by asking participants to make personal judgments about a romantic partner, a parent (young adults) or child (older adults), a close friend, a familiar acquaintance and the self.

Consistent with previous work implicating the default network in mentalizing (Mitchell, 2008; Mar, 2011; Moran *et al.,* 2012; Cassidy *et al*., 2013; Andrews-Hanna *et al.,* 2014), we predicted that both age groups would engage core default network nodes while making trait judgments about known others. Critically, however, as aging is associated with reduced modulation of default network interactivity based on task context (Spreng and Schacter, 2012; Turner and Spreng, 2015; Rieck *et al.,* 2017; see Spreng and Turner, 2019, for a review), we expected age differences in the modulation of default network interactivity during trait judgments across levels of social closeness. With regard to specific patterns of network interactions, we previously reported differential default network coupling with salience network brain regions during mentalizing about close compared to more distal social others in young adults (Laurita *et al.,* 2017). However, a hallmark of neurocognitive aging is over-recruitment of lateral prefrontal brain regions during cognitive tasks (Grady, 2012), potentially reflecting greater reliance on control processes to support performance at lower levels of cognitive demand (Park and Reuter-Lorenz, 2009). Further, prefrontal and default network brain regions are functionally coupled and poorly modulated by task demand in older adults (Amodio and Frith, 2006; Heatherton *et al.,* 2006; Turner and Spreng, 2015; Rieck *et al.,* 2017). Based on these findings, we predicted that for older (but not younger) adults, default network engagement during the mentalizing task would show a dedifferentiated pattern, with greater functional connectivity to lateral prefrontal brain regions across all levels of social closeness.

Alternatively, changes in the structure of social networks in older adulthood suggest a competing hypothesis. Socioemotional selectivity theory (Carstensen, 2006; English and Carstensen, 2014) and the conceptually related convoy theory (Antonucci *et al.,* 2014) posit that the structure of social relationships fundamentally changes in later life. Preferences for closer social bonds (Carstensen, 2006) or age-related functional limitations (Antonucci *et al.,* 2014) result in smaller, more intimate social networks (Bruine de Bruin *et al.,* 2019). This sharper boundary between close and distant social others would result in greater differences in executive control necessary to instantiate more *vs* less frequently accessed mental representations. Contrary to the dedifferentiation hypothesis, age-related changes in social network composition would be associated with more differentiated patterns of functional connectivity between default and executive control networks.

## Materials and methods

### Participants

Participants were 29 healthy, right-handed young adults (16 females, 13 males; *M* age = 24 years, s.d.= 3.5 years) and 27 healthy older adults (12 females, 15 males; *M* age = 67 years, s.d. = 6 years), with normal or corrected-to-normal visual acuity. Participants had no history of psychiatric, neurological or other medical illness that could compromise cognitive functions. Additionally, older adults scored over 26 on the Mini-Mental State Exam (MMSE; Folstein *et al.,* 1975) and were screened for depression with the standardized Geriatric Depression Scale and retained for the current study if ratings ≤1.0 (Yesavage *et al.,* 1983). In accordance with the Institutional Review Board of Cornell University, participants gave written informed consent prior to study enrollment. Across both age groups, study participants were selected for the scanning procedure based on the criterion of being in a long-term, committed, exclusive romantic relationship, defined as being together for ~2 years or more, a critical timepoint for adult romantic attachment formation (Hazan *et al.,* 1991; Fraley and Davis, 1997). The additional recruitment criterion of having a living, adult child was put in place for the recruitment of older adults. Results from the young adults indicating differential patterns of neural activation across social other conditions, specifically recruitment of the salience network in romantic partner and self representations, have been previously reported (Laurita *et al.,* 2017), as have behavioral and neuroimaging results for one specific condition (parent/child) in young and older adults (Laurita *et al.,* 2019a).

### Assessment of self-reported closeness

Participants completed a pre-scan assessment which included specific questions about their various personal relationships as well as standardized self-report measures (WHOTO/IOS). Participants first provided one name per social closeness condition: romantic partner (both young and older adults), parent (young adults) or child (older adults), close friend (both young and older adults) and familiar acquaintance (both young and older adults), in response to prompts (see Laurita *et al.,* 2017 for survey prompts). They then provided the length of their relationship with those individuals. Next, participants completed a self-report measure of attachment (WHOTO; Hazan *et al.,* 1991; Fraley and Davis, 1997). The WHOTO (Hazan *et al.,* 1991; Fraley and Davis, 1997) is an attachment functions measure that determines the individuals with whom participants have attachment relationships. Items are based on four attachment features (two items each): proximity seeking, separation distress, safe haven and secure base. Participants list up to 4 most important figures in their lives for each of the 10 questionnaire items—8 total attachment feature items and 2 general, attachment-related items. The WHOTO can be used in various ways to measure individuals’ attachment to others. In the present study, we utilized it as a continuous measure of attachment with romantic partners, parent and friends by scoring each item based on the individual’s ranking (highest scores = listed first) and totaling these scores; therefore higher WHOTO total scores were indicative of greater levels of attachment.

We also investigated perceived closeness using the Inclusion of Other in Self (IOS) scale (Aron *et al.,* 1992). This scale is a single-item pictorial measure of closeness and interconnectedness in dyads. The seven instances of two overlapping circles of the IOS range from mutually exclusive to highly overlapping in appearance. The IOS is a direct self-report measure of perceived closeness with relationship partners, as it is a visual representation of how individuals think of others and themselves. This measure was included in the pre-scan assessment for each of the following conditions: romantic partner, parent/child, close friend and acquaintance. For an extended discussion of the pre-scan survey and the full survey instrument, see Laurita *et al.* (2019b).

### Behavioral task and fMRI design

**Fig. 1:**
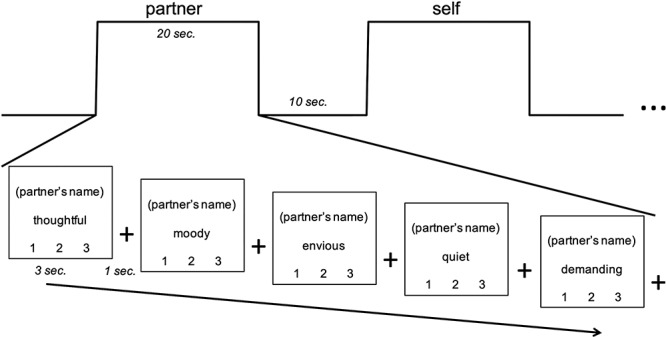
Paradigm, involving trait judgment task for social others. Scale responses corresponded to 1 (unlike this person) to 3 (very much like this person).

During fMRI scans, we used a trait judgment task (Grigg and Grady, 2010; Laurita *et al.,* 2017), exemplified in Figure 1. Participants were asked to think about several individuals in their lives mentioned by name in the pre-scan survey. Participants across both age groups were trained identically, through verbal instructions and a practice block, before entering the scanner. Participants were specifically encouraged to ‘hold the person in mind’ when his/her name appeared on the screen. Each trial contained a trait adjective and a target person’s name; participants rated the target on each trait adjective, on a scale from 1 (unlike this person) to 3 (very much like this person). Blocks comprised five trials, all referring to the same person, in which participants were instructed to continuously hold the target in mind while evaluating this person on each of the five adjectives. Blocks were interleaved with 10 s of fixation to a cross; participants were instructed during training as follows: ‘When you see the fixation cross, focus on the cross and clear your mind’. A motor control condition block was included, in which participants were prompted with ‘Which number?’, showing ‘1’, ‘2’ or ‘3’ on the screen, and were instructed to respond by pushing the button corresponding to the number shown. This particular control was chosen as it is a simple task for assessing baseline button-press response; it utilizes the same three buttons on the button box; it is non-social, in comparison to the social closeness conditions; and it is consistent with prior block designs of mentalizing (e.g. Grigg and Grady, 2010), allowing for comparability between studies.

The experiment consisted of 5 runs, each consisting of 14 blocks, with 350 trials in total. Trials were 3 s long, with a 1 s crosshair fixation screen separating each trial. There were two blocks per run for each of the seven social closeness conditions: partner, parent (for young adults) or child (for older adults), close friend, familiar acquaintance, famous person, self and ‘which number’ control. The order of conditions within each run was randomized. Each task run lasted 7 minutes and 40 s. Blocks were counterbalanced within runs, and the five runs were counterbalanced for each participant to eliminate any possibility of ordering effects of social closeness or trait adjectives. Button-press responses were monitored both in training and in scanning sessions to ensure that responses were made within the 3-s time frame. The famous person condition was excluded from subsequent analyses and interpretation due to numerous participant reports of uncertainty in performing this portion of the task (e.g. the difficulty of bringing an unfamiliar other to mind clearly and responding with a button-press within the several seconds allotted).

Fifty trait adjectives were selected for the study from a list of popularly used personality terms (Anderson, 1968). Adjectives were chosen at random but balanced for valence, with an equal number of positive and negative adjectives. The trait adjectives were presented in a fixed order across blocks, such that each trait adjective was paired exactly once with each social other.

### MRI acquisition and pre-processing

Brain imaging data were acquired using a 3T GE Discovery MR750 MRI scanner with a 32-channel head coil at the Cornell Magnetic Resonance Imaging Facility in Ithaca, New York. Anatomical scans were acquired using a T1-weighted volumetric MRI magnetization prepared rapid gradient echo (TR = 7.7 ms; TE = 3.4 ms; 7° flip angle; 1.0 mm voxels with no gap, 176 slices). Five 7 m 40 s experimental runs of blood–oxygen level-dependent (BOLD) functional scans were acquired with a T2*-weighted multi-echo imaging pulse sequence (TR = 2000 ms; TEs = 12.7, 27.5 and 43 ms; 77° flip angle; 33 axial slices; matrix size = 64 x 64; field of view (FOV) = 240 mm; 33 axial slices; 3.8 mm thick slices).

BOLD fMRI data were pre-processed to correct for motion, physiological noise and scanner artifacts using Multi-Echo Independent Components Analysis (ME-ICA; Kundu *et al.,* 2012, v3.0, https://bitbucket.org/prantikk/me-ica/src/v3/). Each acquired echo was minimally pre-processed before being combined via a voxelwise weighted average to generate an optimal combination time series that preserved the highest T2* weightings for each voxel across the scan. This optimal combination image was decomposed using independent component analysis (fastICA), and the resulting components were categorized as BOLD or noise/non-BOLD based on their T2* decay trajectories. This ME-ICA processing pipeline has been shown to robustly de-noise the fMRI data by removing non-BOLD components (see Kundu *et al.,* 2017 for review; Kundu *et al.,* 2012; Lombardo *et al.,* 2016).

During pre-processing, BOLD fMRI images were normalized to a custom young–old population template derived from 50 younger (25 female; *M* = 22 years, s.d.= 3 years) and 50 older (25 female; *M* = 67 years, s.d. = 7 years) adults. Template participants were selected from an in-house brain bank for low trait motion, as recent work has indicated that trait motion can bias structural scans (mean framewise displacement (FD) = 0.09; Savalia *et al*., 2017). The template can be found here: https://zenodo.org/record/3575255. Anatomical images for template participants were affine registered to MNI space using *@toMNI_Awarp* before being non-linearly, iteratively aligned using *@toMNI_Qwarpar* in AFNI. Data were then smoothed with an 8 mm full width at half maximum (FWHM) Gaussian kernel.

### Partial least squares fMRI analysis

#### Task activation

Task-based analyses were performed using partial least squares (PLS), a multivariate functional neuroimaging analysis technique used to identify whole-brain patterns of activity or connectivity that are associated with tasks (McIntosh *et al.,* 2004; Krishnan *et al.,* 2011). PLS identifies a set of orthogonal latent variables (LVs) that optimally relate BOLD signal with the experimental design. The statistical significance of the detected brain response patterns is assessed through permutation testing, whereas reliability is determined in an independent step by iterative bootstrap resampling with replacement. Because these analyses are performed across voxels in a single step, no correction for multiple comparisons is required.

PLS is a data-driven approach that is sensitive to a distributed voxel response, rather than the activity of individual voxels *per se*. It assesses the covariance between brain voxels (BOLD signal) and the experimental design to identify a limited number of orthogonal components (LVs) that optimally relate brain voxels and experimental design. Using PLS, we were able to examine robust patterns of activity only associated with the experimental conditions (i.e. social closeness). Along these same lines, PLS is capable of analyzing multiple conditions simultaneously to examine covariance of response across experimental conditions.

A mean centered PLS analysis was run in order to examine task-based activity across the whole brain. Activity for each voxel was averaged across blocks for each social closeness condition and normalized relative to activity at fixation preceding the trait judgment. The data matrix was expressed as a voxel-by-voxel deviation from the grand mean across the entire experiment, which was decomposed using singular value decomposition to derive the LVs representing task contrasts. Each brain voxel was given a singular value weight, known as a salience (akin to a component loading in principle component analysis). This value is proportional to the covariance of voxel activity with the task contrast represented by each LV. Multiplying the salience by the BOLD signal value in that voxel and summing the product across all voxels gives a composite brain activity score for each participant on a given LV. We then used these brain scores to examine similarities and differences in brain activity for social closeness conditions and the age groups. Greater activity in brain areas with positive (or negative) weights on a specific LV yields positive (or negative) mean brain scores for a given condition. PLS results can be interpreted as identifying co-varying sets of brain regions in which activity is reliably associated with the specific condition-wise contrasts represented by each LV.

#### Task-related functional connectivity

Task-related functional connectivity analyses were run in order to assess connectivity of dmPFC and vmPFC, due to these regions’ known role in mediating social cognition, also as a function of social closeness. These functional connectivity analyses were performed using seed PLS (McIntosh *et al.,* 2004; Krishnan *et al.,* 2011). Seed PLS examines whole-brain functional activity that correlates with activity in a specified seed region. In seed PLS, LVs represent a decomposition of the covariance between activity in the seed and in all other brain voxels. Since the resultant LVs of seed PLS can identify multiple patterns of functional connectivity, this technique uniquely enables assessment of large-scale brain networks. In two separate seed PLS analyses, activity was first extracted from regions of interest (including peak voxels in the present dataset and 26 neighboring voxels) in dmPFC and vmPFC (MNI coordinates: −6, 54, 30 and − 2, 50, −18, respectively). This extracted activity was correlated across all other brain voxels and across all participants. PLS was then implemented to examine how patterns of correlation differed between social closeness conditions and between age groups.

For the seed-based PLS analyses, the same set of resampling techniques were applied as described above for the task-based PLS. The significance of each LV was determined using 500 permutations with random reordering of the social closeness conditions for each participant. PLS is recalculated for each permutation sample, and the frequency in which the permuted singular value exceeds the observed singular values is determined and expressed as a probability. In a second independent step, the reliability of the saliences for the brain voxels across participants, characterizing each pattern identified by an LV, was determined by bootstrap resampling with replacement, using 100 iterations, to estimate the standard errors for each voxel. We set a minimum bootstrap ratio (conceptually similar to a *Z*-score) at 2.58 equivalent to *P* < 0.01.

## Results

### Behavioral results: assessment of closeness

Our first analyses examined two critical measures: reported attachment status (WHOTO) and perceived closeness (IOS) between self and romantic partners, parents/children and friends, respectively. Table 1 presents descriptive statistics for these measures.

**Table 1:** Descriptive statistics for self-report measures

Measure	Romantic partner	Parent/child	Close friend	Acquaintance
WHOTO *(M,* s.d.*)* out of 40	Young: 32.83, 6.80	Young: 22.31, 8.46	Young: 6.97, 7.56	Young: 0, 0
	Older: 36.70, 6.59	Older: 17.04, 9.65	Older: 4.70, 5.59	Older: 0, 0
IOS *(M,* s.d.*)* out of 7	Young: 4.83, 1.23	Young: 2.93, 1.39	Young: 2.97, 1.55	Young: 1.55, 0.69
	Older: 5.22, 1.45	Older: 3.52, 1.50	Older: 2.89, 1.53	Older: 2.30, 1.38
Length of relationship *(M,* s.d.*)* in years	Young: 3.66, 2.45	Young: 22.90, 4.81	Young: 6.96, 5.49	Young: 4.20, 4.01
	Older: 36.29, 12.42	Older: 29.27, 2.44	Older: 29.15, 13.30	Older: 29.88, 16.37

Note: WHOTO, self-report measure of attachment figures; IOS, Inclusion of Other in Self, self-report measure of perceived closeness

We initially tested for interactions between age and social closeness by condition (partner, parent/child, friend), running separate omnibus multivariate ANOVA (MANOVA) tests for WHOTO scores and IOS scores. There was a significant interaction between condition and age, (*F*(3, 52) = 3.28, *P* = 0.028; Wilk’s Λ = 0.84). We further explored the effect of age in this interaction with *post hoc t*-tests comparing young and older adults, within each condition. Older compared to young adults showed higher attachment as measured by the WHOTO to their partners (*t*(1, 54) = 4.68, *P* = 0.035). In contrast, young adults showed higher attachment to their parents than older adults to their children (*t*(1, 54) = 4.74, *P* = 0.034). However, neither of these differences remained significant after correcting for multiple comparisons. No differences were observed between the age groups for friends *t*(1, 54) = 1.60, *P* = 0.211). Lastly, results of the second MANOVA indicated that there was no significant interaction between condition and age (*F*(3, 52) = 1.421, *P* = 0.25; Wilk’s Λ = 0.92) for perceived closeness (IOS scores).

The MANOVA tests across WHOTO scores and IOS scores also indicated condition differences for young and older adult groups, separately. Results for young adults showed a significant difference between means of romantic partner, parent and friend WHOTO scores (*F*(2, 29) = 22.14, *P* < 0.001). Results of non-parametric analyses mirrored these ANOVA results, as a Friedman test yielded significant differences among repeated measures χ^2^ (2, *N* = 29) = 40.55, *P* < 0.001. We conducted this non-parametric test to account for alternative perspectives that consider WHOTO scores as ordinal data. There was also a significant difference between means of romantic partner, parent and friend IOS scores for young adults (*F*(2, 29) = 68.00, *P* < 0.001).

Results for older adults showed a significant difference between means of romantic partner, child and friend WHOTO scores (*F*(2, 27) = 27.96, *P* < 0.001). Results of non-parametric analyses mirrored these ANOVA results, as a Friedman test yielded significant differences among repeated measures χ^2^ (2, *N* = 27) = 44.24, *P* < 0.001. There was also a significant difference between means of romantic partner, child and friend IOS scores for older adults (*F*(2, 27) = 28.17, *P* < 0.001). Overall, these results confirmed that partner, parent/child and friend represented differing levels of perceived closeness and attachment, for both young and older adults.

### fMRI results: task activation

A mean centered task PLS analysis investigated differences in neural activity between young and older adults and between all social other conditions (partner, parent or child, friend and acquaintance), the self and the motor control condition. This task PLS analysis revealed two significant patterns of activity or LVs.

**Fig. 2:**
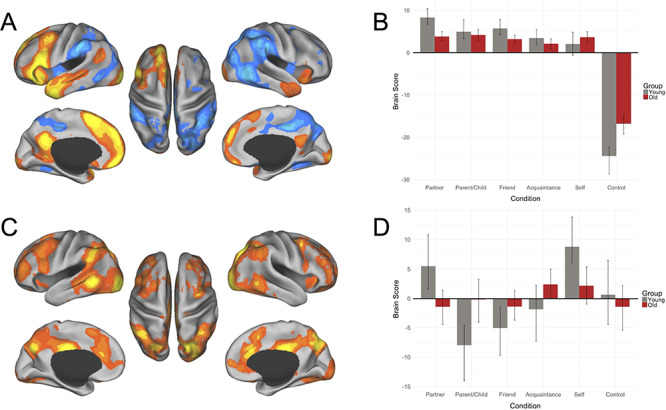
Results of the task PLS analysis. (A) LV1 activation map, (B) LV1 brain scores, (C) LV2 activation map and (D) LV2 brain scores. PLS analysis for young (gray bars) and older (dark red bars) adults contrasted activity across partner, parent or child, close friend, familiar acquaintance, self and control conditions. Warm colors (shades of orange and yellow) on activation maps correspond to positive brain scores, shown by the plotted bars above zero. Cool colors (shades of blue) on activation maps correspond to negative brain scores, shown by the plotted bars below zero. Brain scores represent the cross product of the group result image and the individual subject BOLD response for each given LV. For activation maps, (left) lateral and medial views of left hemisphere, (center) dorsal view, (right) lateral and medial views of right hemisphere. Error bars represent 95% confidence intervals.

The first significant LV accounted for 68.37% of the crossblock covariance (*P* = 0.002). This LV separated all social other representations from the motor control condition for both age groups. This result replicated previous findings that have implicated the default network in mentalizing about others (Krienen *et al.,* 2010; Mar, 2011). We found significant activations for this first LV within the dmPFC, vmPFC, PCC, inferior frontal gyrus, superior frontal gyrus, occipital pole, temporal pole, cerebellum, superior temporal sulcus (STS), angular gyrus, fusiform gyrus, middle cingulate gyrus, retrosplenial cortex, hippocampus and head of caudate (Figure 2 and Table 2).

**Table 2:** Peak activation coordinates, LV1 (results of whole-brain task PLS analysis)

Region	MNI coordinates			
	*x*	*y*	*z*	BSR
Social > control				
Dorsomedial prefrontal cortex	-8	54	32	-16.44
Inferior frontal gyrus	-56	24	12	-15.44
Superior frontal gyrus	-4	14	54	-14.84
Posterior cingulate cortex	-6	-54	30	-12.55
Occipital pole	-22	-98	0	-12.02
Temporal pole	-44	6	-38	-11.59
Cerebellar Crus I	24	-82	-32	-11.46
Ventromedial prefrontal cortex	-2	50	-18	-11.23
Superior temporal sulcus	-48	-36	-2	-7.31
Temporal pole	48	10	-34	-6.97
Angular gyrus	-46	-62	24	-6.70
Fusiform gyrus	-38	-44	-22	-5.03
Middle cingulate gyrus	-2	-14	36	-4.71
Retrosplenial cortex	18	-48	4	-4.49
Hippocampus	-24	-28	-6	-4.12
Cerebellar Crus I/Crus II	-32	-82	-36	-3.83
Inferior frontal gyrus	36	22	18	-3.47
Head of caudate	18	10	14	-3.42
Control > social				
Middle cingulate gyrus	10	-36	44	12.57
Superior parietal lobule	34	-48	44	10.50
MT+	52	-58	-8	10.45
Superior parietal lobule	-38	-42	42	9.43
Cerebellar VIIB	-18	-74	-46	8.91
MT+	-52	-60	-6	8.86
Insula	-40	-10	-4	8.45
Ventral precentral sulcus	50	2	8	7.89
Dorsolateral prefrontal cortex	36	40	24	7.35
Frontal eye field	24	8	56	6.61
Frontal pole	44	40	4	6.56
Dorsolateral prefrontal cortex	-30	30	34	6.42
Inferior temporal gyrus	-52	-32	-22	6.40
Cerebellar VIIIB	18	-58	-52	6.24
Superior frontal gyrus	-22	6	56	4.84
Frontal pole	22	68	-2	3.74

The second significant LV, accounting for 13.65% of the crossblock covariance (*P* = 0.002), showed distinct patterns of activation for young and older participants. In young adults, romantic partner and self conditions were differentiated from parent and friend conditions. Activations in the anterior insula, dorsal anterior cingulate cortex (dACC), posterior middle cingulate, precuneus, occipital pole, supramarginal gyrus, middle frontal gyrus, posterior middle temporal gyrus, inferior frontal gyrus, superior frontal gyrus, frontal pole, thalamus, orbitofrontal cortex, inferior temporal gyrus, head of caudate and precentral gyrus were associated with partner and self representations in the young. Also, in the young participants, the acquaintance and motor control conditions did not contribute to the multivariate pattern of activity evidenced by the CIs crossing zero. In contrast, older participants showed a lack of differentiation in patterns of neural response across all conditions and, as such, did not contribute to the multivariate pattern displayed by young adults (Figure 2 and Table 3).

**Table 3:** Peak activation coordinates, LV2 (results of whole-brain task PLS analysis)

Region	MNI coordinates			
	*x*	*y*	*z*	BSR
For young only: partner and self > parent and friend				
Precuneus	-8	-68	38	6.71
Occipital pole	-22	-96	-4	6.68
Supramarginal gyrus	46	-40	32	6.56
Posterior middle cingulate	8	-24	32	6.54
Middle frontal gyrus	40	8	54	5.79
Posterior middle temporal gyrus	52	-50	-4	5.27
Supramarginal gyrus	-42	-48	34	5.21
Inferior frontal gyrus	-60	18	12	4.98
Dorsal anterior cingulate cortex	10	30	26	4.95
Superior frontal gyrus	16	12	52	4.77
Frontal pole	-40	48	10	4.48
Thalamus	24	-28	0	4.45
Orbitofrontal cortex	-14	30	-24	3.89
Inferior temporal gyrus	-58	-16	-40	3.85
Head of caudate	-12	22	-6	3.84
Anterior insula	30	18	-8	3.54
Precentral gyrus	62	16	14	3.36
Anterior insula	-24	24	-6	3.27

### fMRI results: task-related functional connectivity of mPFC

**Fig. 3:**
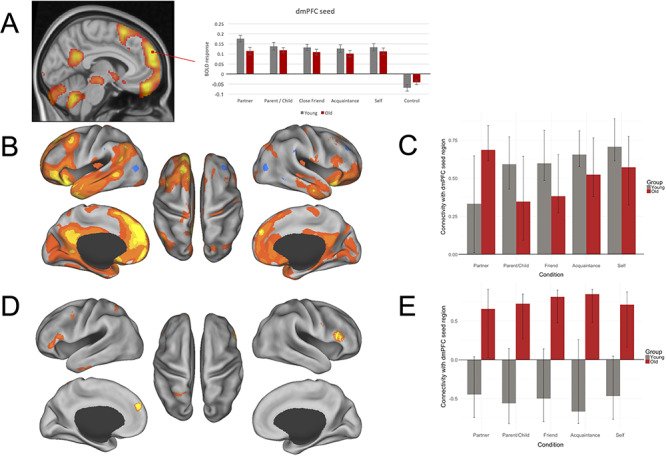
Functional connectivity results for dmPFC. (A) ROI examining a peak activation seed region within dmPFC. Significance is shown through colors within the bar graphs; gray plotted bars correspond with young adult significant response intensities; red plotted bars correspond with older adult significant response intensities. (B–E) Results of dmPFC seed PLS. (B) LV1 connectivity map; (C) LV1 condition- and group-wise correlations, with 95% confidence intervals, between the dmPFC seed region and the whole-brain pattern of connectivity; (D) LV2 connectivity map; (E) LV2 condition and group correlations, with 95% confidence intervals, between the dmPFC seed region and the whole-brain pattern of connectivity. PLS analysis for young (gray bars) and older (dark red bars) adults contrasted connectivity across partner, parent or child, close friend, familiar acquaintance and self conditions. Correlations represent the relationship between brain scores and activity within the dmPFC seed for each condition. Brain scores represent the cross product of the group result image and the individual subject BOLD response for each given LV. For connectivity maps (B) and (D), warm colors (shades of orange and yellow) on connectivity maps correspond to positive brain scores, shown by the plotted bars above zero. Cool colors (shades of blue) on connectivity maps correspond to negative brain scores, shown by the plotted bars below zero. (Left) Lateral and medial views of left hemisphere. (Center) Dorsal view. (Right) Lateral and medial views of right hemisphere.

**Fig. 4:**
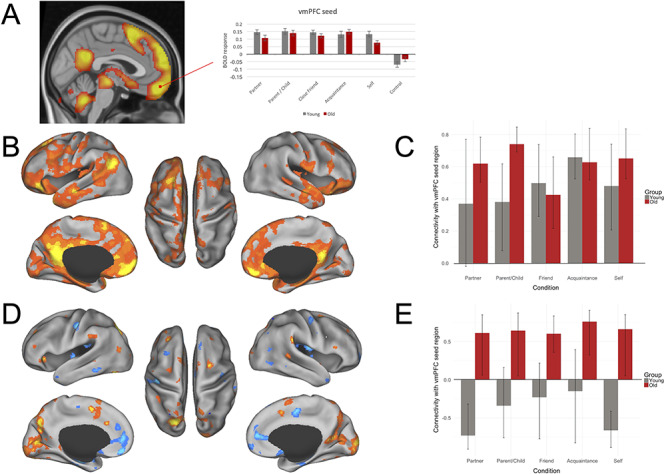
Functional connectivity results for vmPFC. (A) ROI examining a peak activation seed region within vmPFC. Significance is shown through colors within the bar graphs; gray plotted bars correspond with young adult significant response intensities, and red plotted bars correspond with older adult significant response intensities. (B–E) Results of vmPFC seed PLS. (B) LV1 connectivity map; (C) LV1 condition- and group- wise correlations, with 95% confidence intervals, between the vmPFC seed region and the whole brain pattern of connectivity; (D) LV2 connectivity map; (E) LV2 condition and group correlations, with 95% confidence intervals, between the vmPFC seed region and the whole-brain pattern of connectivity. PLS analysis for young (gray bars) and old (dark red bars) contrasted connectivity across partner, parent or child, close friend, familiar acquaintance and self conditions. Correlations represent the relationship between brain scores for each condition and activity within the vmPFC seed for each condition. Brain scores represent the cross product of the group result image and the individual subject BOLD response for each given LV. For connectivity maps (B) and (D), warm colors on connectivity maps (shades of orange and yellow) correspond to positive brain scores, shown by the plotted bars above 0. Cool colors on connectivity maps (shades of blue) correspond to negative brain scores, shown by the plotted bars below 0. (Left) Lateral and medial views of left hemisphere. (Center) Dorsal view. (Right) Lateral and medial views of right hemisphere.

Consistent with our task-based results (Figure 2A), the mPFC has been implicated in self- and other-related processing for both younger and older adults (Moran, Jolly, and Mitchell, 2012). Next we investigated the functional connectivity of mPFC in two seed-based PLS analyses using peak activations from the task analysis (LV1) in dmPFC (Figure 3) and vmPFC (Figure 4).

#### dmPFC connectivity

The seed PLS analysis for dmPFC revealed two significant LVs (Figure 3): a main effect common to both age groups (LV1; Panels B and C) and an age interaction (LV2; Panels D and E). LV1 accounted for 70.21% of the crossblock covariance (*P* < 0.001). This LV demonstrated a shared pattern of connectivity across both young and older adults and across all social other conditions, within the default network; the dmPFC seed showed robust coactivation with PCC, vmPFC and STS. Other significant regions functionally connected with dmPFC for this LV were observed in the middle temporal gyrus, precentral gyrus, posterior superior frontal sulcus, collateral sulcus, lateral occipital cortex, occipital pole, cerebellum, inferior temporal sulcus, ACC, insula, inferior precentral sulcus, postcentral gyrus and middle frontal gyrus.

LV2 accounted for 10.05% of the crossblock covariance (*P* < 0.01) and showed a pattern of connectivity that differentiated older from young adults. Older adults demonstrated a pattern of functional connectivity between the dmPFC seed region and the inferior frontal gyrus bilaterally, medial premotor cortex, lateral occipital cortex, vmPFC, middle temporal gyrus, parahippocampus, precentral gyrus, frontal pole, occipital pole and cerebellum. Connectivity with the dmPFC seed in young adults did not contribute to the multivariate pattern of activity for this LV.

#### vmPFC connectivity

The seed PLS analysis for vmPFC revealed two significant LVs (Figure 4): a main effect common to both age groups and social other conditions (LV1; Panels B and C) and an age interaction (LV2; Panels D and E). LV1 accounted for 73.03% of the crossblock covariance (*P* = 0.002). This LV demonstrated a shared pattern of connectivity across both young and older adults. Activity in the vmPFC seed was positively correlated primarily with a set of brain regions closely overlapping the default network including the PCC/precuneus, hippocampus, middle temporal gyrus, posterior middle frontal gyrus, thalamus, temporal pole, cerebellum, superior frontal gyrus, posterior supramarginal gyrus as well as anterior and orbital PFC and the brain stem. Other areas functionally connected to vmPFC in LV1 included ACC as well as primary visual and visual association regions. This pattern of connectivity with the vmPFC seed was stronger for older *vs* young adults in the romantic partner and parent/child conditions.

LV2 accounted for 7.56% of the crossblock covariance (*P* < 0.034) and again showed a pattern of connectivity that differentiated older from young adults. Older adults demonstrated greater connectivity of the vmPFC seed to dorsolateral PFC regions as well as precuneus and dorsal ACC. Other regions that showed significant functional connectivity with vmPFC for older adults included the occipital fusiform gyrus, supramarginal gyrus, occipital pole, frontal orbital cortex, cerebellum, superior frontal gyrus, frontal pole, intracalcarine cortex and lateral occipital cortex. In contrast, young adults in the romantic partner and self conditions demonstrated greater connectivity between the vmPFC and dmPFC, hippocampus, ACC, insula and caudate. Other regions that demonstrated greater functional connectivity with vmPFC for these conditions in young adults were the subcallosal cortex, postcentral gyrus, parietal and central operculum cortices, supplementary motor area, temporal pole, amygdala, frontal pole, inferior temporal gyrus, brain stem, parahippocampus, middle temporal gyrus, superior frontal gyrus and precentral gyrus. Connectivity with the vmPFC seed in young adults in the parent, close friend or acquaintance conditions did not contribute to the multivariate pattern of activity for this LV.

## Discussion

We investigated age-related differences in patterns of brain activation and functional connectivity during mentalizing about known others, stratified by levels of social closeness. Consistent with predictions, we observed engagement of the default network, critically involved in mentalizing, during trait judgments of close others in both young and older adults. However, age-related differences emerged in the modulation of whole-brain activation patterns across levels of social closeness. For young adults, default and salience network activity was observed during judgments of close (self, partner) but not more distal (friend, acquaintance) social others (see Laurita *et al.,* 2017 for an extended discussion). This pattern of default network modulation based on social closeness did not emerge for older adults (Figure 2C and D).

We next examined functional connectivity of two core default nodes from the whole-brain analyses that have been strongly implicated in mentalizing (LV 1, Figure 2A and B; see also Amodio and Frith, 2006; Heatherton *et al.,* 2006; Mitchell *et al.,* 2006). Analyses revealed greater within default network coupling for young adults. In contrast, older adults showed greater coupling with lateral PFC and other cognitive control regions. These findings suggest that social judgments about known others depends on interactions between default network regions and other large-scale brain systems, and these connectivity patterns are modulated by degree of social closeness and differ by age.

### Network modulation of social representations

For both age groups, a pattern of increased activation in dmPFC, vmPFC and PCC was observed across all social closeness conditions, but not the motor control condition (Figure 2A and B). This pattern of results provides critical evidence that our social task engaged default network brain regions in both age groups and these brain patterns were not confounded by basic visuomotor demands of the task. These brain regions form part of the ‘social brain’ (Mitchell, 2008) and are also core nodes of the default network (Buckner *et al*., 2008; Andrews-Hanna *et al.,* 2014; Uddin *et al.,* 2019). A second pattern emerged in the whole-brain analysis, showing default network interactions with the salience network in young adults. Critically, this pattern differentiated brain response during mentalizing about oneself or a romantic partner *vs* a parent/child or acquaintance. These results suggest that in young adulthood default network engagement alone may be necessary but insufficient to instantiate the full spectrum of relationships in our social milieu. The salience network plays a role in the detection of behaviorally relevant environmental stimuli, including internally generated or remembered information as well as the processing of personally relevant inputs (Uddin, 2015; Uddin *et al.,* 2019). While speculative, judgments about oneself or a chosen life partner may engage different levels of salience processing beyond that of an age-different (parent or child) or low-familiarity (acquaintance) other. However, there are myriad determinants of how social closeness is reflected that limit our ability to test this possibility directly here (see Thornton and Mitchell, 2017).

Of greater relevance for the current study, older adults did not express the second, whole-brain pattern showing default–salience covariance of activity during social judgments (Figure 2C and D). This suggests that older adults may not modulate these ‘social brain’ regions based on differences in social closeness, as observed in young adults. We know that mental representations become increasingly semanticized in later life as fluid abilities decline (Park *et al*., 2001; Spreng and Turner, 2019). For example, in older adulthood personal (or autobiographical) recollections of events from one’s past include fewer discrete episodic details and greater gist-based recollections (Levine *et al.,* 2002; Spreng *et al.,* 2018). It is possible that social representations undergo a similar transformation with age, resulting in less distinct representations of social relationships and a corresponding reduction in the distinctiveness of neural representations of social others. The altered structure of social networks in older age (Antonucci *et al.,* 2014; English and Carstensen, 2014; Bruine de Bruin *et al.,* 2019) does not appear to result in a differentiated pattern of network interactivity based on social closeness. Our task paradigm, however, was not designed to test this alternate hypothesis directly as we did not include a manipulation or measure of age differences in the frequency of mentalizing about distant *vs* close social others. The interaction between shifting social structures and mental representations of close others represents an important avenue for future research.

To further interrogate the possibility that neural representations of social others become less distinct with advancing age, we next examined the impact of age and social closeness on the functional connectivity patterns for two core default network nodes (vmPFC and dmPFC) that emerged from the whole-brain analyses and which have been consistently implicated in mentalizing about social others (Gobbini *et al.,* 2004; Krienen *et al.,* 2010; Moran *et al.,* 2012; Hassabis *et al.,* 2014; Thornton and Mitchell, 2017). Based on our previous work, we hypothesized that trait judgments for social others would be associated with connectivity between cognitive control and default network brain regions in older but not younger adults.

### Default network interactivity and social other representations in older adulthood

Consistent with our whole-brain findings, and with previous research implicating the default network in mentalizing and social cognition (Krienen *et al.,* 2010; Mitchell *et al*., 2008), both dmPFC and vmPFC nodes were functionally connected with other default network brain regions for all levels of social closeness in both age groups (Figures 3B, C and 4B, C). Critically, both dmPFC and vmPFC analyses revealed a second significant pattern of functional connectivity that was reliable only in the older adult group. For both seed regions, older adults demonstrated functional connectivity with prefrontal regions bilaterally (Figures 3D and 4D). A pattern of increased recruitment of brain regions implicated in cognitive control and reduced suppression of the default network has been commonly observed in neurocognitive aging studies (Grady, 2012). We and others have shown that these processes may be coupled and are poorly modulated by task context in older adults (Spreng and Schacter, 2012; Turner and Spreng, 2015; Rieck *et al.,* 2017; Spreng and Turner, 2019). Here the dmPFC was functionally coupled with more ventral aspects of lateral PFC bilaterally. This pattern corresponds to social working memory experiments in young adults, where parametric increases in cognitive control demands for social information resulted in increased activity in dmPFC and lateral PFC regions (Meyer *et al.,* 2012). Further, increases in lateral PFC activity were also observed at lower levels of cognitive control demand in older *vs* young adults (Cappell *et al.,* 2010; Turner and Spreng, 2012). In the context of these earlier findings, we suggest that social trait judgments for known others may impose greater cognitive control demands for older than young adults, resulting in greater age-related functional coupling of these regions.

The vmPFC seed-based analysis also revealed a second LV associated with all social judgments in older adults, as well as self and partner judgments in young adults. Robust positive connectivity was observed between vmPFC and the superior medial supplementary motor area, a region that has been implicated in control processes in working memory (Braver and Barch, 2006; Turner and Spreng, 2012). The vmPFC seed was also functionally connected to dorsolateral PFC regions bilaterally. We have previously described this pattern as the Default to Executive Coupling Hypothesis of Aging (DECHA, Spreng and Turner, 2019). Specifically, the DECHA proposes that as mental representations become increasingly semanticized, and less differentiated, greater connectivity between default and executive control regions is necessary to engage these representations in the service of ongoing task goals. Consistent with this idea, we have demonstrated that greater default to executive coupling is associated with more semanticized autobiographical memory recall (Spreng *et al.,* 2018). As noted above, we speculate that representations of close others may also become less distinct and more semanticized with age. Consistent with the DECHA, default network brain regions showed greater, and less flexible, coupling with cognitive control regions across levels of social closeness. In contrast, young adults demonstrated a similar pattern to that observed in the whole brain findings: greater connectivity between the vmPFC seed and other default and salience network regions was observed for self and partner judgments. This suggests that default network engagement in trait judgments is modulated by social proximity in young adults.

In sum, we have shown that the neural representation of close others is altered in older *vs* young adults. First, older adults show greater coupling between regions of the ‘social brain’ and cognitive control regions. Second, this coupling is poorly modulated by social context (i.e. the closeness of the social relationship). In contrast, young adults show increased coupling of default and salience-related brain regions. Unlike older adults, this pattern varied by social closeness, with greater interactions among these large-scale brain systems for self and romantic partner judgments. Future research is necessary to explore how this hierarchy of social relationships is represented in the functional neural architecture and how these representations change across the adult lifespan.

### Study limitations

In the current study, we have defined social closeness based on a model of attachment and perceived inclusion of other in the self. However, as noted above, there are multiple dimensions along which closeness may be stratified, including demographic factors, perceived similarity, relationship length, fondness and dyadic relatedness. As suggested by Thornton and Mitchell (2017), representations of close others based on these different schemata may have distinct neural signatures and thus represents an interesting and important line of future research, especially in consideration of how these schemata of closeness may manifest differently across age groups. Another factor potentially influencing perceived closeness across young and older adults is dependence; for example, college-aged young adults may be more financially dependent on their parents and less so on their romantic partners, whereas the reverse may be true for older adults. Although our past work (Laurita *et al.,* 2019a) has noted similar patterns of neural activity supporting how young adults mentally represent their parents and older adults represent their adult children, future investigations could more specifically examine dependence, especially in comparing young and older adults’ romantic relationships.

Further, we elected to investigate neural network dynamics across the continuum of known others, potentially limiting the generalizability of the present findings to unknown others. We explicitly drew this experimental distinction as we were specifically interested in investigating a range of closeness for known others. Adding an anonymous other would have represented a categorical distinction that, while theoretically important, was not a primary focus of the current study. While making trait judgments about known others, older adults may attend more to positive traits than negative traits (Carstensen *et al.,* 1999). This task design may have affected the pattern of results observed. Valence of emotional trait judgments for known others remains an area for future inquiry. Finally, we recognize the limitations of an extreme groups design and the absence of a true lifespan sample. For one, relationship length with partner, close friend and acquaintance was correlated with age and is potentially confounding in the studied participants. Additionally, there may well have been cohort effects within our sample, a limitation which future studies could address by collecting longitudinal data across age decades. However, we continue to pursue this program with the ultimate goal of collecting individuals from each decade of life to investigate the network neuroscience of social cognition and mentalizing across the full adult lifespan.

## Conclusions and implications

Taken together, our findings highlight the importance of investigating neural network dynamics in the study of neurocognitive aging. In fact, our findings suggest that well-established network neuroscience models of brain aging (e.g. default-executive coupling) may inform the study of social cognitive aging. The implications of these findings for social functioning in real-world contexts are unknown and will be an important area of future research. For example, we have recently published a review and a model of financial vulnerability in older adulthood that emphasizes the potential role of social cognition in vulnerability to financial exploitation by personally known others (Spreng *et al.,* 2016). Further, we have demonstrated that older adults who were victims of financial exploitation by acquaintances or family members displayed altered default network dynamics as compared to those who had avoided exploitation (Spreng *et al.,* 2017). While speculative, our findings here suggest that altered default network dynamics in older adulthood may be an important marker of social judgment and decision-making in real-world domains, with important implications for functional capacity in later life.
